# The Pivotal Role of Aryl Hydrocarbon Receptor-Regulated Tight Junction Proteins and Innate Immunity on the Synergistic Effects of Postbiotic Butyrate and Active Vitamin D3 to Defense against Microbial Invasion in *Salmonella* Colitis

**DOI:** 10.3390/nu15020305

**Published:** 2023-01-07

**Authors:** Fu-Chen Huang, Shun-Chen Huang

**Affiliations:** 1 Department of Pediatrics, Kaohsiung Chang Gung Memorial Hospital and Chang Gung University College of Medicine, Kaohsiung 833, Taiwan; 2 Department of Anatomic Pathology, Kaohsiung Chang Gung Memorial Hospital and Chang Gung University College of Medicine, Kaohsiung 833, Taiwan

**Keywords:** acyl hydrocarbon receptor, postbiotics, active vitamin D3, tight junction, innate immunity, *Salmonella* colitis

## Abstract

Our recent report illustrated the unitedly advantageous effects of postbiotic butyrate on active vitamin D3 (VD3)-orchestrated innate immunity in *Salmonella* colitis. There is growing awareness that aryl hydrocarbon receptor (AhR) can regulate intestinal immunity and barrier function, through modulating cecal inflammation and junction proteins expression. Hence, we researched the participation of AhR-regulated tight junction functions on the united effects of butyrate and VD3 on intestinal defense to *Salmonella* infection. *Salmonella* colitis model were elicited by oral gavage with 1 × 10^8^ CFU of a *S. typhimurium* wild-type strain SL1344 in C57BL/6 mice. Before and after the colitis generation, mice were fed with butyrate and/or VD3 by oral gavage in the absence or presence of intraperitoneal injection of AhR inhibitor for 4 and 7 days, respectively. We observed that butyrate and VD3 could concert together to reduce the invasion of *Salmonella* in colitis mice by enhancing cecal cytokines and antimicrobial peptides expression and reducing zonulin and claudin-2 protein expressions in mucosal stain, compared to single treatment, which were counteracted by AhR inhibitor. It implies that AhR is involved in the united effects of butyrate and VD3 on the intestinal defense to *Salmonella* infection in colitis mice. This study discloses the promising alternative therapy of combining postbiotic and VD3 for invasive *Salmonellosis* and the pivotal role of AhR pathway.

## 1. Introduction

*Salmonella* spp. are influential Gram-negative food-borne pathogens of humans and animals. If treatment is delayed or inadequate, severe systemic infections may lead to high-mortality complications including meningitis, osteomyelitis, sepsis, and toxic megacolon. A globally rising occurrence of food-borne multi-drug-resistant strains of *S. typhimurium* in human infections has been noted [1,2], which may be associated with increased hospitalization, development of sepsis, treatment failure and additional mortality [3].

Medical nutrition therapy (MNT) is an evidence-based, customized nutrition process intended to help treat certain medical conditions. MNT is based on decades of medical research on the relationship between diet, nutrition, and health outcomes. As chronic diseases become more prevalent, with prolonged and changing lifespans, MNT is a key scientific platform to improve clinical symptoms and reduce inflammation, leading to induction and/or maintenance of disease remission, and finally promote health and prevent diseases. Inventor Thomas Edison said that “the doctor of the future will give no medicine but will interest his patients in the care of the human frame, in diet and in the cause and prevention of disease”.

Accruing evidence shows that nutrition can curb the immune system through metabolites, either produced by microbiota metabolism or by host digestion. Food is not merely a source of nutrients for preserving vital biological functions, it is composed of nutritive constituents that orchestrate immune cell interaction as well. Aryl hydrocarbon receptor (AhR) is a ligand-activated transcription factor that integrates environmental, dietary, microbial, and metabolic cues to control complex transcriptional programs. An important resource of nutritional AhR ligands is microbiota metabolism, that is, postbiotics. Representative examples of postbiotics, short-chain fatty acids (SCFAs) including acetate, butyrate and propionate, derived from the fermentation of dietary fibers by the microbiota, are able to activate AhR signaling.

The role of AhR on colitis and bacterial infection is increasingly brought on stage. The AhR takes part in innate immune responses to microbial invasion of barrier tissues. The AhR can regulate intestinal barrier function, via modifying tight junction integrity and adjustment of junction proteins expression [4,5,6,7]. Explicit deletion of the AhR from intestinal epithelium brings about a weakened response to *C. rodentium* infection [8] and inflammatory damage; therefore, the importance of epithelial AhR expression in intestinal homeostasis and protection cannot be overemphasized. If AhR is deficient in mice, the mice are vulnerable to *Citrobacter rodentium* [9,10,11], which is a natural mouse pathogen widely used to simulating enteropathogenic and enterohemorrhagic *Escherichia coli* (*E. coli*) infections in human [12].

Dietary intervention on maintaining barrier function can contain bacteria invasion. SCFAs facilitate the establishment of intestinal barrier, and shield the intestinal barrier from the disruption of LPS by inhibition of NLRP3 inflammasome and autophagy [13]. They could be used for the recovery of the intestinal epithelial barrier disrupted by several enteric pathogen toxins [14]. Accumulating in vitro and in vivo evidences demonstrate advantageous effects of vitamin D on intestinal permeability [15]; however, study regarding the influence of this vitamin on the tight junction proteins expression in *Salmonella* colitis mice are still lacking.

Previously, we demonstrated the advantageous effects of combining postbiotic butyrate on active vitamin D3 (VD3)-orchestrated innate immunity in *Salmonella* colitis by enhancing antimicrobial peptides (AMPs) but suppressing inflammatory cytokines responses via vitamin D receptor (VDR). However, the role of AhR-mediated tight junction (TJ) protein expression on the benefits of combined butyrate and VD3 on *Salmonella* invasiveness are not yet reported. Therefore, we investigate if AhR orchestrates the effects of combined butyrate and VD3 on cytokines response (IL-17A and IL-22), antimicrobial peptides (LL-37), and tight junction proteins expression to block the invasion of *Salmonella* in colitis mice.

## 2. Materials and Methods

### 2.1. Bacterial Strains

The *Salmonella* wild-type strain *S. enterica* serovar Typhimurium SL1344 (*S*. Tm) was grown for 2 h at 37 °C in the Lysogeny broth supplemented with 50 ug/mL streptomycin, diluted 1:100 in fresh broth, and sub-cultured for 16 h at 37 °C under mild aeration. Then, bacteria were washed twice in PBS and suspend in PBS to 10^9^ CFU/ mL.

### 2.2. Reagents

The butyrate and standard laboratory reagents were obtained from Sigma (St. Louis, MO, USA) or Fisher Scientific (Pittsburgh, PA, USA). The stock solution of 1, 25-dihydroxyvitamin D3 (VD3) (IsoSciences, Ambler, PA, USA) was stored at 22 °C in the dark.

### 2.3. Postbiotics Preparation

Sodium butyrate powder was bought from Sigma-Aldrich (Merck KGaA, Darmstadt, Germany) and stored at room temperature. The dose used for animal experiments was sodium butyrate (100 mg/kg mice). Weight the powder of butyrate and dissolve it in sterile ddH_2_O. After filtered in the solution by 0.45 μm filter, the stock was dispensed in vial and stored at −20 °C for further use.

### 2.4. AHR Inhibitor Solution Preparation

The aryl hydrocarbon receptor inhibitor (CH-223191) powder was purchased from Sigma-Aldrich (Merck KGaA, Darmstadt, Germany) and stored at −20 °C. The powder of AhR inhibitor was weighted, dissolved in DMSO as the stock solution and stored at −20 °C. For preparing the injection solution, dilute the stock in 1× PBS buffer. Moreover, DMSO in 1× PBS buffer was used as vehicle.

### 2.5. Animal Experiments

All mice were obtained from the National Laboratory Animal Center. Animal experiments were approved by the Kaohsiung Chang Gung Memorial Hospital Institutional Animal Care and Use Committee adhering to the legal requirements.

Six- to eight-week-old female C57BL/6 mice were bred in a specific pathogen-free room in the Kaohsiung Chang Gung Memorial Hospital animal center. Mice were divided into the following groups: Control (Open control), ST (*S*.Tm infected), VD (VD3 treated and *S*.Tm infected), BU (butyrate treated and *S*.Tm infected), VD+BU (VD3 plus butyrate treated and *S*.Tm infected), and VD+BU+AHRi (VD3 plus butyrate treated and AhR inhibitor and *S*.Tm infected).

Before and after the colitis induction, mice were oral gavage given VD3 0.2 μg/25 g mice or postbiotics BU or combination of both in the absence or presence of intraperitoneal injection of AhR inhibitor (AHRi) for 4 and 7 days, respectively. Other groups were given 100 μL sterile water (Open control) or 100 μL PBS (*S*.Tm group) and intraperitoneal injection of DMSO solution. Salmonella colitis model were achieved as previously reported [16,17]. On day 14, mice were sacrificed by CO_2_ asphyxiation and the tissue samples from the intestinal tracts, spleens, and livers were collected for analysis. The detail of the protocol was presented in supplementary Figure S1.

Animal and diarrhea situation score and loss of body weight were recorded during the experimental process. The diarrhea situation was scored as follows: 5 = Mice live energetically; 4 = Mice experience diarrhea and pasty stools; 3 = Mice experience loose stools and reduced mobility; 2 = Mice are weak and demonstrate abnormal behavior; 1 = Mice lose their lives. We also calculate the spleen index as the assessment of the immunity.

H&E-stained slides were scored according to the scoring scheme for quantitative pathological analysis of cecal inflammation [18]. Two researchers evaluated the slides separately. Moreover, the combined pathological scores for each tissue sample were determined as the sum of the averaged scores.

### 2.6. Analysis of Salmonella Loads in Spleen and Liver

All aseptically removed tissues from mice were weighing and recorded. Then, the spleen and liver were immersed into room temperature PBS with 1% triton X-100. The spleen and liver were homogenized as in manufacturer’s protocol and previous reports [16,17]. To determine the numbers of *Salmonella* colonized, plating appropriate dilutions on MacConkey agar plates mixed with 50 μg/mL streptomycin for culture under mild aeration at 37 °C for 16 h. The minimal detectable values were 20 CFU/organ in the spleen and 100 CFU/g in the liver. 

### 2.7. Immunohistochemistry (IHC) Staining Procedures

Paraffin sections of paraffin-embedded tissue samples from the cecum of each animal were mounted on glass slides. After deparaffinized and rehydrated, the slides were microwaved in a retrieval buffer for the purpose of antigen retrieval. The slides were then blocked in 10% normal serum with 1% BSA in TBS for 2 h at room temperature. After draining for a few seconds, the slides were then incubated with the primary antibody at 4 °C overnight. Then, the slides were rinsed with TBS buffer for few times. Subsequently, slides were incubated for 1 h at room temperature with secondary antibody (HRP-conjugated antibody). Next, slides were rinsed and incubated with the chromogen (3,3′-diaminobenzidine) to visualize the target protein and counterstained with hematoxylin. Finally, they were dehydrated, cleared, and mounted for further analysis.

### 2.8. Immunohistochemistry Staining Analysis

An automated whole-slide scanning device (3DHISTECH, Sysmex, Switzerland) and software (Pannoramic viewer, Sysmex, Switzerland) were implemented. The scanned images were analyzed by using free software ImageJ Fiji. Semi-quantitative IHC is a powerful method for investigating protein expression within tissues [19]. By using software ImageJ Fiji, we conducted deconvolution and downstream analysis. The area of scanning images which are interested were circled and measured by a trained pathologist. Ten regions of interest were chosen from every slide image and at least three experiments were done to collect the values of images for further statistical analysis.

### 2.9. Quantitative Real-Time PCR Analysis of Cecum or Cultured Cells RNA

Samples of the cecum were immediately snap-frozen in liquid nitrogen after procurement, and stored at −80 °C. Total RNA was extracted from the cecal tissue, using TRI Reagent (Ambio #15596018) and Directzol RNA MiniPrep kit, in line with the manufacturer’s instructions. The RNA was reverse transcribed into cDNA. Then, the quantitative real time PCR and analysis of mRNA levels were performed. The primers for the mouse genes of interest and reaction protocol was set as in previous reports [16,20] except mouse IL-17A, forward, 5′-ATCCCTCAAAGCTCAGCGTGTC-3′, reverse, 5′-GGGTCTTCATTGCGGTGGAGAG-3′; mouse IL-22, forward, 5′-GTCAACCGCACCTTTATGCT-3′, reverse, 5′-CATGTAGGGCTGGAACCTGT-3′; mouse CRAMP, forward, 5′-GCCGCTGATTCTTTTGACAT-3′, reverse, 5′-GCCAAGGCAGGCCTACTACT-3′; and mouse AhR, forward, 5′-ACATCACCTATGCCAGCCG-3′, reverse, GACTTAATTCCTTCAGCGGGGA-3′. The MIQE guidelines were taken into account for the methods and analysis [21].

### 2.10. Statistical Analysis

The statistical analysis was achieved by employing GraphPad Prism 8 software (GraphPad Software, San Diego, CA, USA). For three or more nonparametric variables, we used a Kruskal–Wallis one-way ANOVA to decide the variance. A *p*-value of <0.05 was considered statistically significant.

## 3. Results

### 3.1. The Involvement of AhR in the Synergistic Effects of Admixture of VD3 and Butyrate on the Severity of Salmonella Colitis

**Figure 1:**
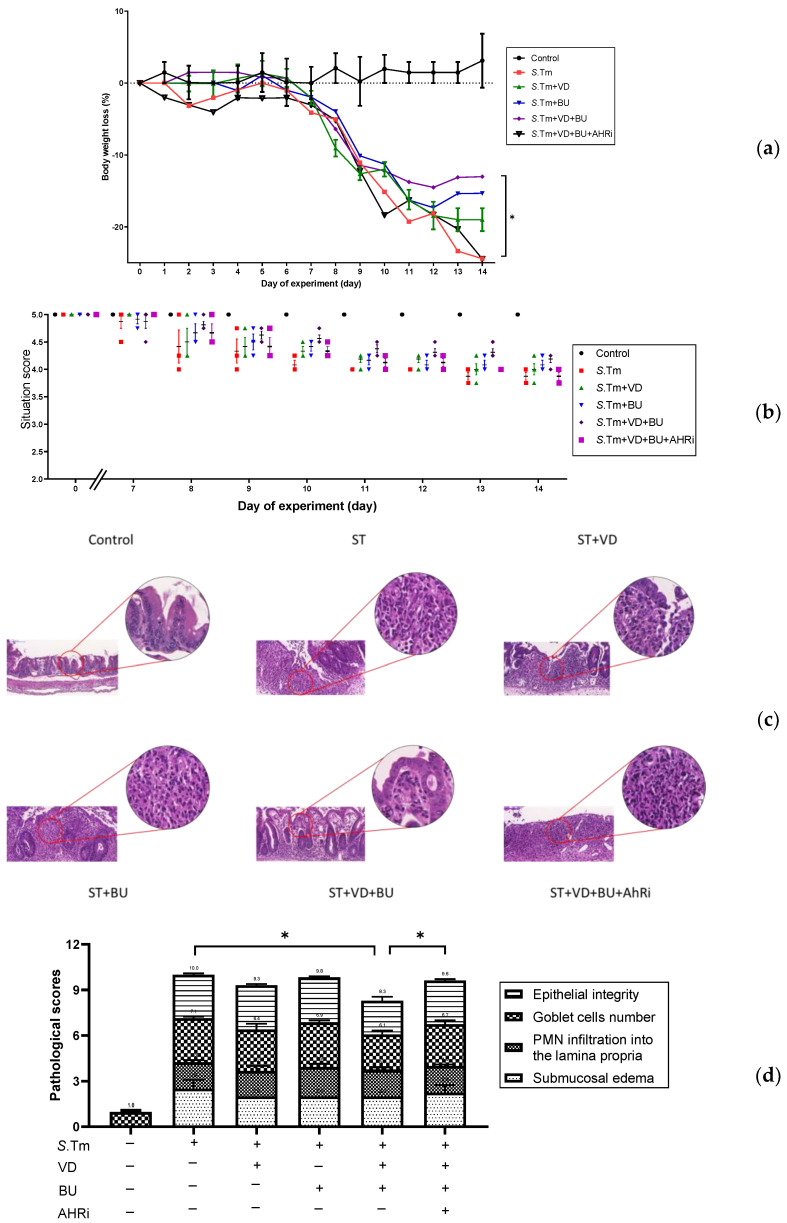
The involvement of AhR in the synergistic effects of admixture of VD3 and butyrate attenuates the severity of Salmonella colitis in mice. Mice were bred and housed under the technical regulation of the animal facility of the Center for Cellular and Biomolecular Research, Kaohsiung, Taiwan. The 6–8-week-old female C57BL/6 mice (Charles River, Wilmington, MA, USA) were treated or infected as described in the material and methods and divided into the following groups: Control (Open control), ST (*S*.Tm infected), VD (VD3 and *S*.Tm infected), BU (butyrate 100 mg/kg mice and *S*.Tm infected), VD+BU (combination of VD3 and butyrate plus *S*.Tm infected), and VD+BU+AHRi (combination of VD3 and butyrate plus *S*.Tm infected and AhR inhibitor). Diarrhea situation scores (**a**) and loss of body weight (**b**) of mice were recoded daily. Segments of cecum were harvested, fixed in formaldehyde, and stained with hematoxylin and eosin. Representative histological images (×20 and ×50 magnification) of cecum from the different experimental groups were shown in (**c**) and the analyzed pathological scores for colitis in (**d**) The data shown are means ± SEM (*n* = 7 mice/group). *, *p* < 0.05.

To assess the involvement of AhR in the synergistic effects of admixture of VD3 and butyrate on the severity of Salmonella colitis, we examined the cecal pathology of SL1344-infected WT mice treated by VD3 or butyrate treatment along with intraperitoneal injection of AhR inhibitor. Consistent with our previous research [18] in the histopathological analysis of H&E-stained cecal sections, we observed obvious pathological colitis in the Salmonella infected WT mice group in Figure 1a. In contrast, we demonstrated that combining BU and VD3 significantly reduce the severity of Salmonella colitis in C57BL/6 mice, including diarrhea, loss of body weight, and pathologic scores. Using the histological scoring system, we found the severity of Salmonella colitis was ameliorated significantly in the combination-treated groups than in the infection-only WT mice (Figure 1b). Furthermore, inhibition of AhR counteracted the benefit of treatment, either alone with VD3 or butyrate or in combination. This suggests that AhR is involved in the synergistic effects of BU on the VD3-mediated reduced severity of Salmonella colitis.

### 3.2. The Involvement of AhR in the Synergistic Effects of Admixture of VD3 and Butyrate on the Cecal Cytokines and Antimicrobial Peptides in Salmonella Colitis Mice

**Figure 2:**
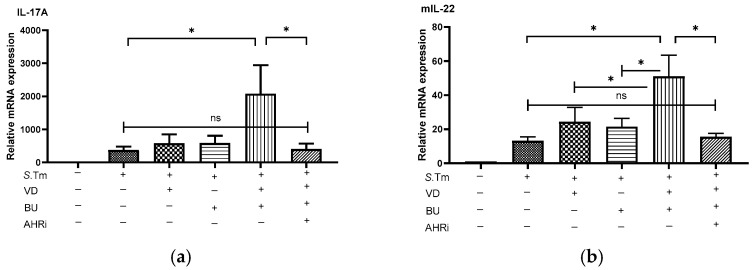

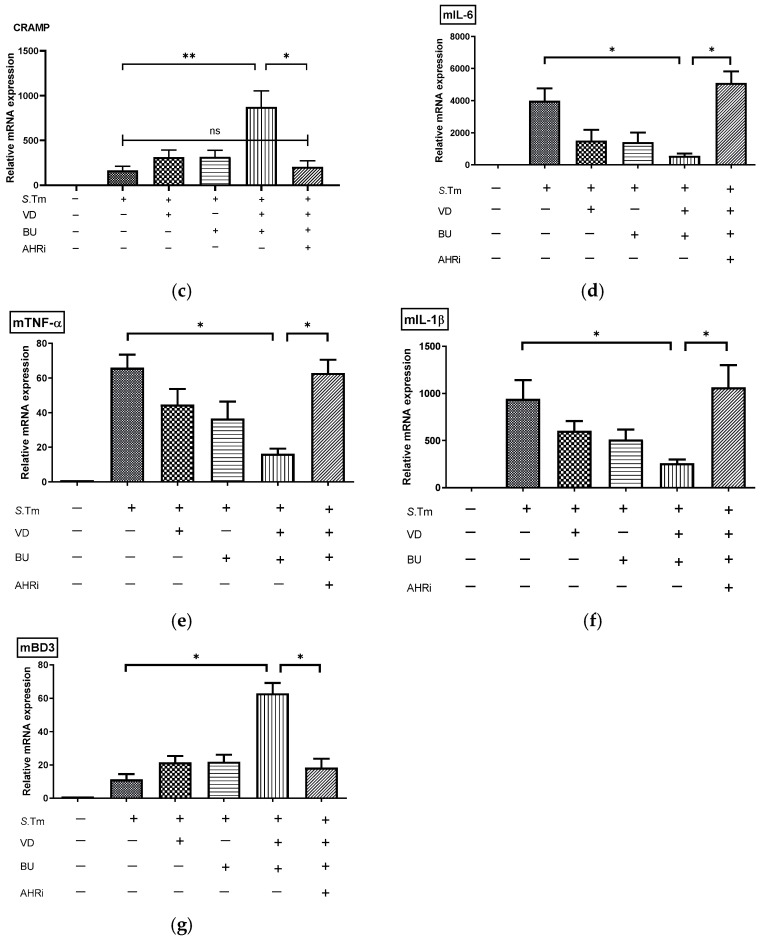
The involvement of AhR in the synergistic effects of BU on the VD3-regulated cecal proinflammatory cytokines and AMPs in *Salmonella* colitis mice. Mice were treated or infected as described in the material and methods. Before and after the colitis induction, mice were oral gavaged with BU or VD3 or combination of both in the absence or presence of intraperitoneal injection of AhR inhibitor (AHRi) for 4 and 7 days, respectively. Total RNA was extracted from the cecal tissues. IL-17A (**a**), IL-22 (**b**), LL-37 (CRAMP) (**c**), mIL-6 (**d**), mTNF-α, (**e**), mIL-1β (**f**), and mBD-3 (**g**) expressions were analyzed using quantitative RT-PCR. Values are measured as fold increase compared to the level of control mice. The data shown are means ± the SEM (*n* = 7 mice/group). * *p* < 0.05, ** *p* < 0.01.

Higher IL-6, IL-1β, and TNF-α levels in response to LPS was observed in macrophages from AhR^–/–^ mice than WT mice [22]. Moreover, upon AhR activation, there are increased production of antimicrobial peptides, IL-10, IL-22, prostaglandin E2, and Foxp3 [23]. It suggests the role of AhR in the suppressive effects of combined butyrate and VD3 on the inflammatory responses and enhancing effect on AMPs in *Salmonella* colitis. To investigate the effects of admixture of VD3 and butyrate on the immune responses in *Salmonella*-infected mice, the gene expression of cytokines and antimicrobial peptide was quantified using real-time PCR in the cecal tissue of infected WT mice in the absence or presence of VD3 or butyrate, along with intraperitoneal injection of AhR inhibitor. Local, cecal gene expression of cytokines and antimicrobial peptide (Figure 2) was induced in *Salmonella*-infected mice. By contrast, inflammatory cytokines were synergistically suppressed (e.g., mIl6, mIl-1β, mTNF-α) in the cecal tissue of *Salmonella*-infected mice treated with admixture of VD3 and butyrate, whereas mIl-17A, mIl-22, and antimicrobial peptides (e.g., mBD-3 and CRAMP) were synergistically enhanced. Furthermore, inhibition of AhR counteracted the effects on mRNA expressions by treatment, either alone with VD3 or butyrate or in combination. This suggests that AhR is involved in the synergistic effects of combined VD3 and butyrate on the local inflammatory responses and antimicrobial peptide in the cecum of *Salmonella*-infected mice.

Altogether, the results suggest that AhR is involved in the synergistic effects of combined butyrate and VD3 on the severity of *Salmonella* colitis, augmenting antibacterial and antiinflammatory responses.

### 3.3. Admixture of VD3 and Butyrate Exerted Reduction of Bacterial Translocation in Salmonella-Infected Mice

**Figure 3:**
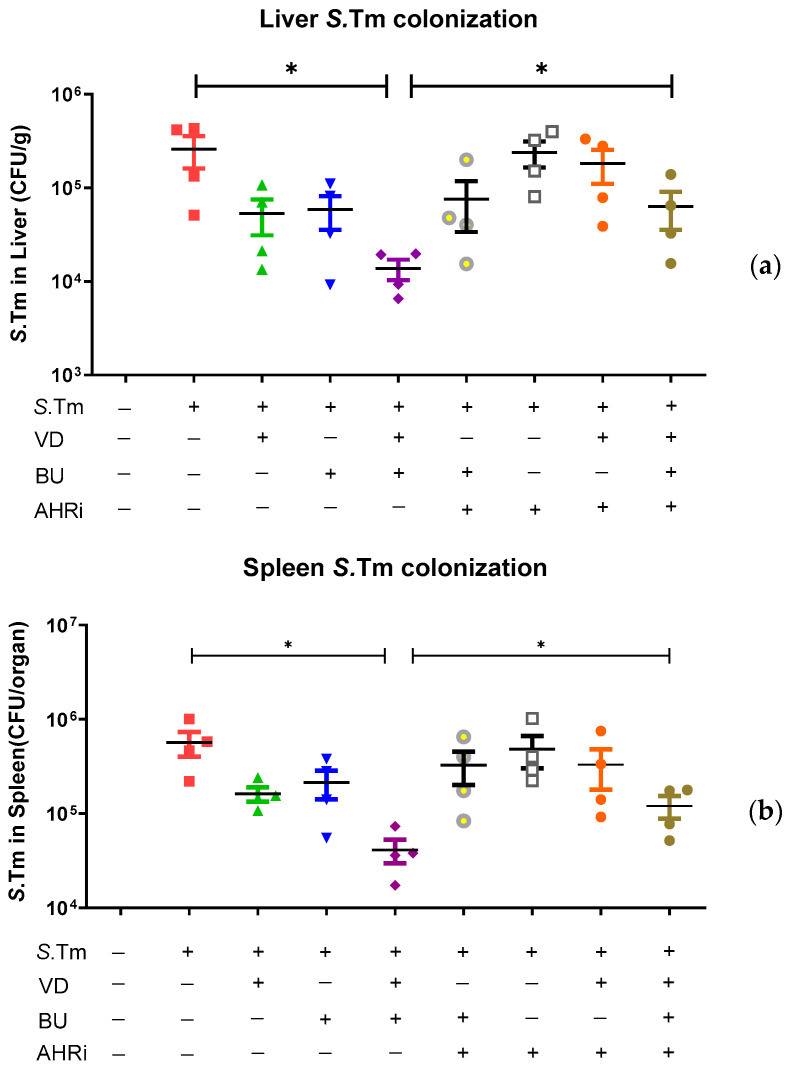
The involvement of AhR in the combined effects of VD3 and butyrate on attenuating systemic bacterial translocation of *Salmonella* colitis mice. Mice were bred and housed under the technical regulation of the animal facility of the Center for Cellular and Biomolecular Research, Kaohsiung, Taiwan. The 6–8-week-old female C57BL/6 mice (Charles River, USA) were treated or infected as described in the material and methods. Before and after the colitis induction, mice were oral gavage with BU or VD3 or combination of both in the absence or presence of intraperitoneal injection of AhR inhibitor (AHRi) for 4 and 7 days, respectively. The number of bacteria was counted from liver (**a**) and spleen (**b**) homogenates of different groups, as shown in the material and methods. The data shown are represented as the means ± the SEM of the bacterial load in the liver and spleen (*n* = 7). * *p* < 0.05.

Previous reports by Khailova et al. [24] and our group [18] revealed that VD3 can reduce systemic bacterial translocation and mortality in experimental sepsis in mice during weanling or *Salmonella* colitis. To explore the impact of combining treatment of butyrate and VD3 on bacterial invasion, tissues of liver and spleen were acquired from *Salmonella* colitis mice untreated or treated with VD3, butyrate or combination of both homogenized and plated on LB plates. The CFU values were estimated. Results demonstrated that combined treatment of butyrate and VD3 exerted reduction of bacterial loads in liver or spleen of *Salmonella* colitis mice (Figure 3).

We observed that the combination of BU and VD3 reduced bacterial colonization in liver and spleen, compared to SL1344 infection only, while AhR inhibitor counteracted the combined effects.

### 3.4. Admixture of VD3 and Butyrate Exerted Synergistic Effect on Tight Junction Proteins Expression in Cecal Mucosa of Mice with Salmonella Colitis

Enteric infections have been implicated in the pathogenesis of several pathological conditions, including allergic, autoimmune, and inflammatory diseases, by causing impairment of the intestinal barrier and alterations in intestinal permeability. The zonulin-driven opening of the paracellular pathway may represent a defensive mechanism, which flushes out microorganisms, thus contributing to the innate immune response of the host against bacterial colonization of the intestine [25]. *Salmonella* infection induced significantly enhanced claudin-2, resulting in an increased bacterial invasion and translocation. Therefore, we investigated the zonulin and claudin-2 protein expression at colon mucosal in *Salmonella* colitis mice model under the treatment of VD3 and butyrate as well as the role of AhR. The proteins expression of zonulin or claudin-2 was analyzed using immunohistochemistry staining on the cecal tissue of *Salmonella* colitis mice in the absence or presence of butyrate or VD3.

**Figure 4:**
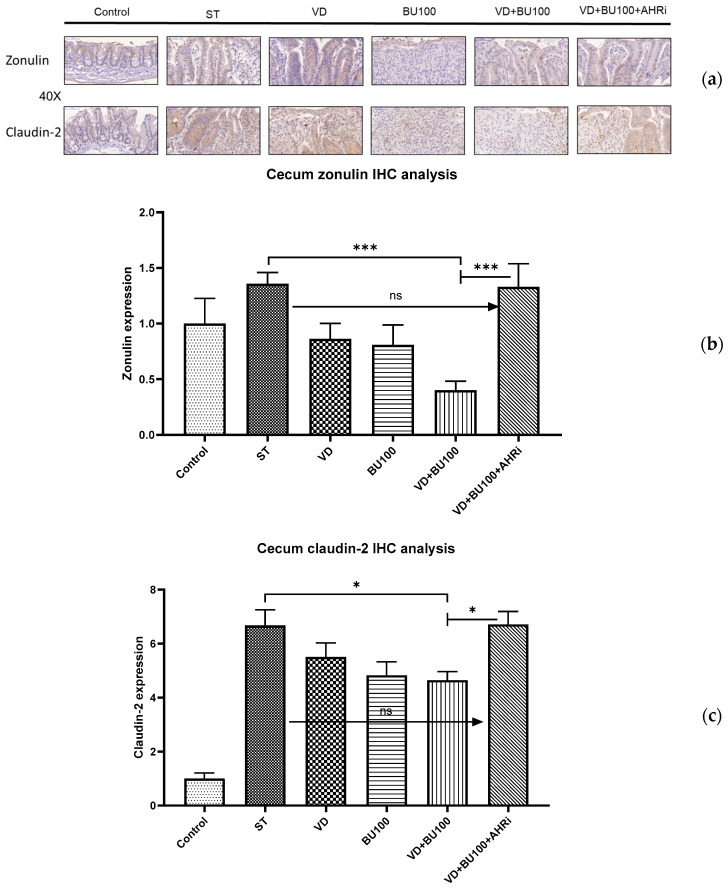
The involvement of AhR in the combined effect of VD3 and butyrate on reducing the colon epithelial zonulin and claudin-2 proteins expression following Salmonella colitis in mice. Mice were treated or infected as described in the material and methods and divided into the following groups: Control (Open control), ST (*S*.Tm infected), VD (VD3 and *S*.Tm infected), BU (butyrate 100 mg/kg mice and *S*.Tm infected), VD+BU (combination of VD3 and butyrate plus *S*.Tm infected), and VD+BU+AHRi (combination of VD3 and butyrate plus *S*.Tm infected and AhR inhibitor). (**a**) Zonulin and claudin-2 proteins expression in these groups were detected by immunohistochemistry staining (original magnification, ×400; scale bar, 25 µm; *n* = 3). The levels of zonulin (**b**) and claudin-2 (**c**) immunohistochemistry staining were analyzed and calculated by image J. * *p* < 0.05, *** *p* < 0.001.

As shown in Figure 4, our results revealed that zonulin and claudin-2 were present at significantly reduced levels in cecal mucosa in *Salmonella* colitis via the treatment of butyrate and VD3, compared with the sham group, whereas inhibition of AhR counteracted the combined effect by both treatments. This suggests that AhR is involved in the synergistic effects of combined VD3 and butyrate on the mucosal zonulin and claudin-2 proteins expression in the cecum of *Salmonella*-infected mice.

## 4. Discussion

We observed that the combination of butyrate and VD3 synergistically reduced the translocation of *Salmonella* to liver and spleen by increasing cecal production of mIL-22, mIL-17a, and mLL-37 mRNA expressions as well as decreasing zonulin and chaudin-2 proteins expression in cecal histologic stain, compared to single treatment. In contrast, AhR inhibitor i.p. counteracted the synergistic effects of butyrate and VD3. This suggests a critical role of AhR on the synergistic effects of butyrate and VD3 on enhancing antibacterial and mucosal barrier in *Salmonella* colitis.

Following ligand binding, AhR is translocated to the nucleus, where it forms a heterodimer with AhR nuclear translocator, thereby inducing AhR-dependent gene expression. Immune cells produce IL-22 in an AhR-dependent way. The IL-22 will then induce IEC to proliferate and produce antimicrobial peptides [26]. IL-22 enhances the innate immunity of tissues by inducing β-defensin 2, β-defensin 3 and S100A7 in epithelial cells [27]. Although AhR is a novel negative regulator of IL-17-mediated signaling and inflammation in vitro [28] and reciprocal regulation of interleukin-17A and interleukin-22 secretion through AhR activation in CD4+ T cells [29], we demonstrated that IL-17A and IL-22 mRNA expression were synergistically enhanced by both treatment in *Salmonella* colitis and inhibition of AhR restored the enhancement of both mRNA expression. Accordingly, IL-17 induces an inflammatory tissue response and is elevated in patients with *Salmonellosis* [30] to suppress invasion of the organism to enteric mucosa by inducing AMPs (e.g., mBD3) [31], whereas IL-22 ameliorates intestinal inflammation [32] and mediates innate immunity to protection against *Salmonella* infection [33]. SCFAs may regulate intestinal inflammation through AhR-mediated decreased IFN-γ, IL-6, IL-12, TNF-α, IL-7, and IL-17, along with reduced microbial translocation in the gut [23]. Moreover, activation of AhR increases the production of IL-10, IL-22, and AMPs. Furthermore, IL-17A plays a critical role in maintaining mucosal barrier integrity by increasing tight junctions [34].

Upon AhR activation in the gut, there is improvement of epithelial barrier [23]. AhR maintain intestinal permeability and ameliorating DSS-induced colitis [35]. The epithelial barrier function is regulated by direct and indirect mechanisms of AhR activation. When AhR is activated, there are inhibition of TNF-α/IFN-γ-induced decrease in TJ disruption [5] or increased TJ protein expression in response to IL-22 [36]. Therefore, AhR constrains pro-inflammatory pathways in intestinal epithelial cells (IECs), and thus, preserves intestinal permeability.

Altered intestinal permeability, a component of the intestinal barrier, plays a role in many pathological conditions [37]. Butyrate makes a contribution in activating hypoxia-inducible factor (HIF) in the hypoxic region of the colon, consequently promoting intestinal epithelial barrier function, antimicrobial defense, and mucus production [38]. Vitamin D-treated IECs showed increased TEER, with upregulation of ZO-1, occludin, and several claudins [39], while VDR-deleted mice showed increased colonic permeability and susceptibility to DSS-induced colitis [39]. The protective role of VD3 and VDR was shown in the mucosal injury and epithelial TJs disruption with reduced intestinal permeability and severity in DSS-induced acute colitis [40]. Administration of vitamin D to mice in DSS-colitis model [41] shored the expression of TJ proteins, enhanced barrier function, and decreased intestinal permeability and circulating levels of LPS, leading to improved colitis symptoms.

Additionally, the production of antimicrobial cathelicidin, such as LL-37, is modulated by butyrate or propionate in colonocytes [42]. Butyrate was shown to augment levels of LL-37 in Caco-2 and HT-29 cells [43]. The probable associations of LL-37 in host protection were disclosed by Raqib et al. [44], who showed that butyrate upregulated the expression of CAP-18, the rabbit homologue to LL-37, which is critical for protection against *shigella* infection. Aside from cathelicidin, acetate, propionate, butyrate, as well as phenyl derivatives of butyrate, were able to enhance β-defensin 2 and β-defensin 3 expression in a porcine-derived colon cell line [45]. This finding was further elucidated by the finding [46] that butyrate was capable of arousing the expression of β-defensin 2 and β-defensin 3 in the colon and ileum of pigs, which eventually gave rise to protection against severe infection with *E. coli.* LL-37 expression was relying on butyrate activation of MEK–ERK pathway in the human colon cancer cell line SW620 [42], whereas p38/MAP kinase displays little effect on AMP production [42]. C-Jun N-terminal kinase (JNK) also positively regulates phenylbutyrate-induced cathelicidin production in tissue epithelial cells [47]. Moreover, GPR43 mediated the SCFAs-induced expression of AMPs via activation of STAT3 and mTOR [48].

AhR is a ligand-activated transcription factor that is vital for intestinal homeostasis by suppressing inflammation and by sustaining the epithelial barrier in the gastrointestinal tract [49], a rich source of AhR ligands, and to protect the gut upon infection or induced colitis. AhR activation lightens *E. coli*-induced mastitis by strengthening tight junction protein expression and limiting NF-κB pathway activation [50]. Intestinal barrier dysfunction has been associated in the pathogenesis and progression of septicemia. The tight junction proteins expression is changed in an experimental model of sepsis [51]. Zonulin-dependent intestinal barrier impairment is an early step leading to altered gut permeability and increased morbidity/mortality in the DSS colitis model [52]. Zonulin is the only known physiological modulator of intercellular tight junctions described so far that is involved in immune/tolerance response balance. Loss of barrier function caused by upregulation of zonulin brings about an uncontrolled influx of dietary and microbial antigens. The two key ignitions of zonulin release are bacteria and gliadin. Several enteric pathogens, including *Salmonella typhi* and *E. coli*, have been made known to cause zonulin release from the intestine [25]. In patients with septicemia, an increase in their serum zonulin levels was observed [53]. When patients were critically ill, their low vitamin D serum level (less than 20 ng/dL) correlated with increased plasma zonulin and endotoxin concentration [54]. Treatment with the zonulin inhibitor AT-1001 markedly diminished the severity of IL-10 knockout colitis [55]. We observed inhibition of AhR increase zonulin expression, suggesting that AhR exerts suppressive effect on zonulin expression of colon and AhR-mediated decreased zonulin expression in colon tissue may explain the reduced severity of *Salmonella* colitis and invasiveness in liver and spleen.

During intestinal inflammation, Claudin-2 acts as a mediator of leaky gut barrier [56]. AhR activation by FICZ relieved colonic inflammation, reduced IL-6 and claudin-2 expression, and kept intestinal barrier function in a mouse model of DSS-induced colitis [6]. An in vitro study also demonstrated that AhR ligand plays a protective effect on IL-6 induced disruption of intestinal epithelial barrier function through repressing the expression of claudin-2. In the same colitis mouse model, AHR ablation leads to susceptibility to bacterial infection due to disruption of tight junction [5]. *Salmonella* infection considerably boosted Claudin-2 expression, leading to a beneficial environment for bacterial invasion and translocation [57]. A lack of VDR regulation results in a stout increase of Claudin-2 at the mRNA and protein levels post-infection [57]. In DSS-treated VDR-/- mice, Claudin-2 was significantly increased. Furthermore, in ulcerative colitis patients, the inflamed intestine had low VDR and increased Claudin-2. Claudin-2 and autophagy regulator ATG16L1 are VDR target genes [58]. The ability of VD3 on inhibiting inflammatory cytokine levels and downregulating claudin-2 protein which was upregulated in active UC patients [59], suggests that vitamin D may signify a potential therapeutic agent for the treatment of active UC. Butyrate also impedes permeability-promoted claudin-2 tight junction protein expression through an IL-10RA-dependent mechanism [60] to promote epithelial barrier function.

From multiple in vitro and in vivo studies, several cytokines have been shown to mediate claudin-2 transcriptional regulation, including IL-6, TNF-α, IL-17A, and IL-22 [61,62,63]. IL17A promotes antimicrobial or epithelial barrier genes such as β-defensins, claudin [64], and zona occludens 1. Enterohemorrhagic *E. coli* (EHEC) infection of C57Bl/6J mice showed markedly augmented claudin-2 expression that correlated with increased intestinal permeability [65]. TNFα has well characterized roles in modulating TJ permeability [66].

### Limitations

The concept and study are novel and deserve to do further mechanistic investigations, such as the following: (1) how vitamin D and butyrate influence each other in upregulating AhR; (2) how molecules/cytokines (such as VD3, BU, AhR, IL-22, and IL-17a) can ameliorate *Salmonella*-induced colitis by modulating mucosal immunocytes (T cells, B cells, and monocytes/macrophages); (3) the role of AhR-mediated autophagy on cross talk between IECs and macrophage to promote intestinal barrier function. These are all important issues to clarify the pivotal role of AhR in the mechanism of the *Salmonella* colitis and find out the better solution to defense the disease. Furthermore, mechanistic experiments using VDR-knockout (KO) and AhR-KO mice will make the story clearer than inhibitors; however, literature on this topic is currently not available in Taiwan.

## 5. Conclusions

We observed that the combination of butyrate and VD3 synergistically upregulated cecal cytokines mIL-17A and mIL-22, and the antimicrobial peptides mLL-37 (CRAMP) and mBD-3 mRNA expressions in *Salmonella* colitis mice, compared to single treatment, while reducing inflammatory cytokines, including Il-1β, IL-6, and TNF-α. Moreover, the combination synergistically reduced the tight junction proteins zonulin and claudin-2 expression in cecal tissue and bacterial colonization in liver and spleen. Inhibition of AhR abrogated the synergistic effects by the combined treatment, either on cecal mRNA and tight junction proteins expression, as well as bacterial colonization. This suggests that AhR is involved in the synergistic effects of combined butyrate and VD3 on the invasiveness of *Salmonella* infection in colitis mice by enhancing antibacterial responses to defense against infection but reducing tight junction proteins expression to block invasiveness. These findings will not only explore how butyrate and vitamin D can strengthen the human body’s innate immunity against invasion of *Salmonella* infection, but also the critical role of AhR on the combined effects of postbiotics and VD3. Therapies aimed at enhancing AhR activity, reversing the pathological effects of zonulin, restoring gut integrity, optimizing an effective immune response represent exciting avenues of discovery, and potential therapeutics for critically ill patients in the future. Globally, it will certainly contribute to *Salmonella* infection control, and the same theory could be applied to various pathogens; additionally, an extension of the therapeutic strategy may well be applied to the research of other infections.

## Data Availability

The data presented in this study are available on request from the corresponding author.

## Supplementary Materials

The following supporting information can be downloaded at: https://www.mdpi.com/article/10.3390/nu15020305/s1, Figure S1: protocol for experiment.
