# Supplementary figures and images for: The Pivotal Role of Aryl Hydrocarbon Receptor-Regulated Tight Junction Proteins and Innate Immunity on the Synergistic Effects of Postbiotic Butyrate and Active Vitamin D3 to Defense against Microbial Invasion in Salmonella Colitis
Source: Nutrients. 2023 Jan 7;15(2):305. doi: 10.3390/nu15020305 (PMC9860786; doi:10.3390/nu15020305)

## Slide 1
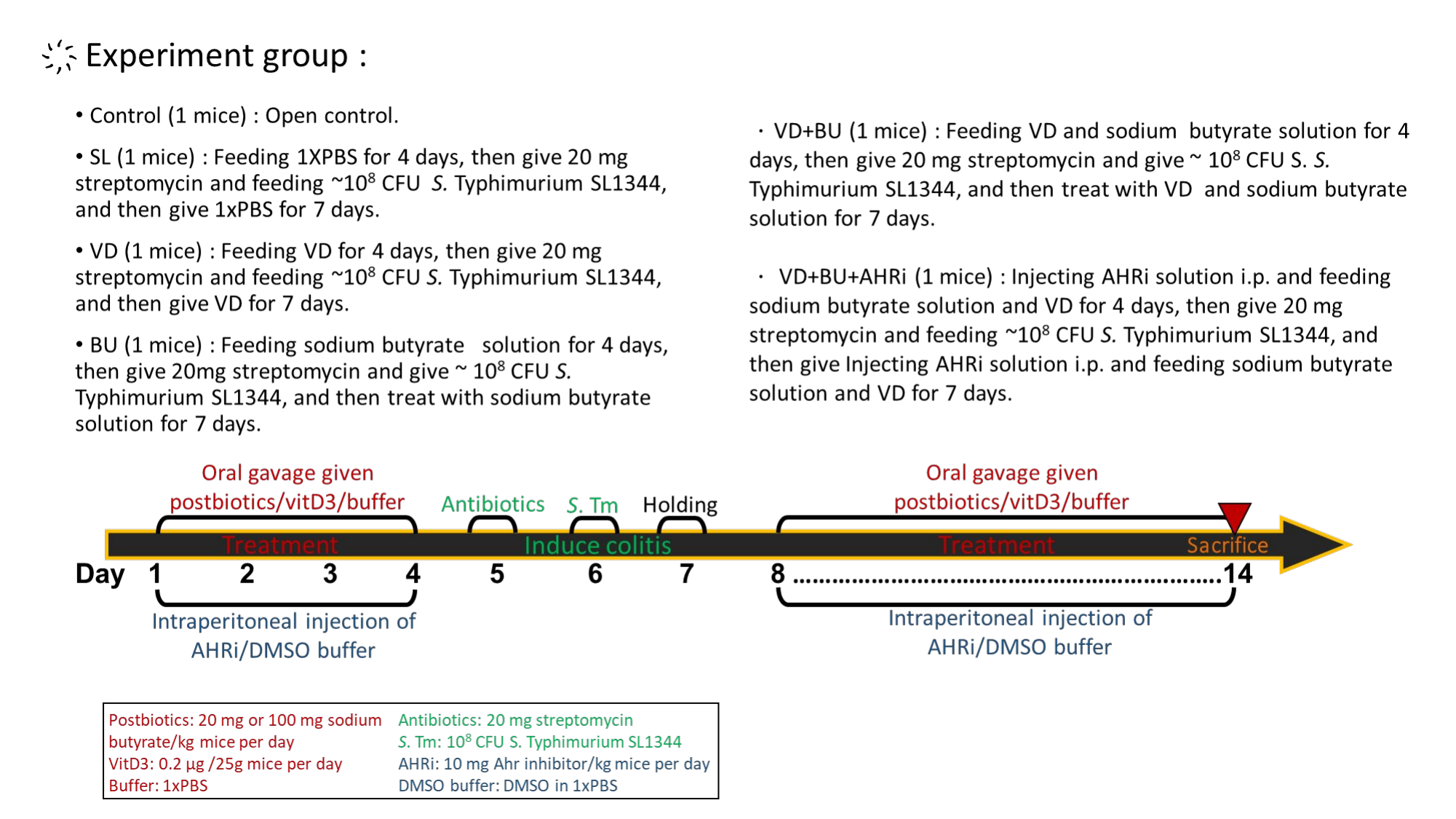

Supplement: Supplementary file 1 [file nutrients-15-00305-s001.zip › nutrients-2089256-supplementary.pptx]
